# Establishment of a reverse transcription real-time quantitative PCR method for Getah virus detection and its application for epidemiological investigation in Shandong, China

**DOI:** 10.3389/fmicb.2022.1009610

**Published:** 2022-09-23

**Authors:** Xinyu Cao, Xiangshu Qiu, Ning Shi, Zhuo Ha, He Zhang, Yubiao Xie, Peng Wang, Xiangyu Zhu, Wenxin Zhao, Guanyu Zhao, Ningyi Jin, Huijun Lu

**Affiliations:** ^1^Changchun Veterinary Research Institute, Chinese Academy of Agricultural Sciences, Changchun, Jilin, China; ^2^College of Veterinary Medicine, Northwest A&F University, Yangling, Shaanxi, China; ^3^College of Animal Sciences, Institute of Preventive Veterinary Medicine, Zhejiang University, Hangzhou, Zhejiang, China; ^4^Key Laboratory of Zoonoses Research, Ministry of Education, Institute of Zoonosis, College of Veterinary Medicine, Jilin University, Changchun, China

**Keywords:** GETV, *Nsp1*, RT-qPCR, ROC curve, epidemiological investigation

## Abstract

Getah virus (GETV) is a mosquito-borne, single-stranded, positive-sense RNA virus belonging to the genus *Alphavirus* of the family *Togaviridae*. Natural infections of GETV have been identified in a variety of vertebrate species, with pathogenicity mainly in swine, horses, bovines, and foxes. The increasing spectrum of infection and the characteristic causing abortions in pregnant animals pose a serious threat to public health and the livestock economy. Therefore, there is an urgent need to establish a method that can be used for epidemiological investigation in multiple animals. In this study, a real-time reverse transcription fluorescent quantitative PCR (RT-qPCR) method combined with plaque assay was established for GETV with specific primers designed for the highly conserved region of GETV *Nsp1* gene. The results showed that after optimizing the condition of RT-qPCR reaction, the minimum detection limit of the assay established in this study was 7.73 PFU/mL, and there was a good linear relationship between viral load and *Cq* value with a correlation coefficient (*R*^2^) of 0.998. Moreover, the method has good specificity, sensitivity, and repeatability. The established RT-qPCR is 100-fold more sensitive than the conventional RT-PCR. The best cutoff value for the method was determined to be 37.59 by receiver operating characteristic (ROC) curve analysis. The area under the curve (AUC) was 0.956. Meanwhile, we collected 2,847 serum specimens from swine, horses, bovines, sheep, and 17,080 mosquito specimens in Shandong Province in 2022. The positive detection rates by RT-qPCR were 1%, 1%, 0.2%, 0%, and 3%, respectively. In conclusion, the method was used for epidemiological investigation, which has extensive application prospects.

## Introduction

GETV is a single-stranded positive-sense RNA virus transmitted by *Culex* and *Aedes* mosquitoes and belongs to the genus *Alphavirus* of the family *Togaviridae* (Murphy et al., 2008). The genome contains 2 open reading frames (ORFs) which are responsible for encoding non-structural proteins (*NSP*)*1* to *NSP4* and structural proteins, including *Cap*, *E3*, *E2*, *6K,* and *E1,* respectively (Powers et al., 2001). GETV has first discovered in 1955 in Culex caught in Malaysia (Morita and Igarashi, 1984) and subsequently spread widely across Eurasia and the transpacific region.

Natural infection of GETV has occurred in a variety of vertebrates, including humans (Marchette et al., 1978), but currently, only swine, horses, bovines, and foxes have clinical symptoms of the disease. GETV infection causes fever, diarrhea, abortion, and stillbirth in sows and neurological symptoms or death in piglets (Kuwata et al., 2018; Xing et al., 2020). Moreover, clinical signs in infected swine will develop into viremia, so swine is considered to be the main amplifying host of GETV. Horses are important amplifying and circulating hosts for GETV (Bannai et al., 2015), and infection with GETV may result in fever, generalized rash, and leg edema (Nemoto et al., 2015). In 2018, GETV was isolated from cattle with sudden onset of fever for the first time in the world (Liu et al., 2019; Gao et al., 2020). Sequence analysis showed that this strain was highly consistent with the strain isolated from local mosquitoes and a swine herd in Hunan Province, China, which demonstrated that a mosquito-borne swine GETV could infect and circulate in bovines. GETV infection in blue foxes was first detected in 2017 (Shi et al., 2019) and led to fever, anorexia nervosa, and even death in foxes, suggesting a further expansion of the GETV infection spectrum. In addition, the identification of neutralizing antibodies against GETV from human serum showed that the antibody titers in fever patients were significantly higher than that of healthy individuals (Hu et al., 2012). As a widespread arbovirus, the epidemic scope and host numbers of GETV are expanding (Shi et al., 2021). What’s more, in China, the transmission of GETV has been detected in 18 provinces so far (Lu et al., 2020), which has already caused serious economic losses to local farms. Its lethality to economic animals has posed a serious threat to animal husbandry and public health worldwide (Lu et al., 2020).

At present, the laboratory detection methods of GETV mainly include virus isolation and identification (Shibata et al., 1991), serological diagnosis, and virus nucleic acid detection. However, the virus isolation method needs to be performed in the P2 laboratory, which is time-consuming and laborious. Serological identification is more sensitive than virus isolation. But there may be cross-reactivity between the *Alphaviruses*, which reduces the accuracy of the method. Up till the present moment, most of the research has been directed toward nucleic acid testing, which is easier to perform and more sensitive. Nucleic acid detection methods of GETV have been developed including RT-PCR (Ogawa et al., 2009; Nemoto et al., 2015), randomly amplified polymorphic cDNA (cDNA RAPD; Hu et al., 2012), reverse transcription loop-mediated isothermal amplification (RT-LAMP; Liu et al., 2019), and RT-qPCR (Sam et al., 2018; Shi et al., 2018; Xia et al., 2021). Among them, RT-qPCR has gradually become a popular virus detection method due to its low sample requirement and excellent experimental repeatability, sensitivity, and specificity (Schmittgen and Livak, 2008). What’s more, SYBR Green I RT-qPCR is more economical for the detection of a large number of samples.

In this study, an SYBR Green I RT-qPCR method for the highly conserved *Nsp1* gene region of GETV was innovatively proposed. In contrast to the traditional method of quantification by constructing standard plasmids, this study combines plaque assay to quantify infectious virus particles. Based on ROC curve analysis, this method has a high diagnostic value, and the best cutoff value was obtained. The method was successfully used for the epidemiological investigation of GETV in Shandong Province, China.

## Materials and methods

### Virus strains and clinical samples

The strain used to establish the RT-qPCR assay is GETV JL17/08 (GenBank accession number MG869691). Chikungunya virus (CHIKV), Japanese encephalitis virus (JEV), porcine reproductive and respiratory syndrome virus (PRRSV), Akabane virus (AKAV), equine influenza virus (EIV), Sindbis virus (SINV), and Zika virus (ZIKV) kept in the laboratory were used to detect the specificity of the RT-qPCR method. One hundred and forty-three serum samples from horses serologically identified as strongly positive for GETV, stored in the laboratory, were used for ROC curve analysis. A total of 2,847 clinical serums derived from swine (1106), horses (597), bovines (667), and sheep (477) with about 17,080 mosquito specimens in 171 pools were collected in 2022 from Heze, Jining, Yantai, Qingdao, and Binzhou in Shandong Province, China.

### Virus amplification and titration

Vero-E6 cells were cultured in DMEM medium containing 10% fetal bovine serum. GETV JL17/08 was inoculated into Vero-E6 monolayer cells at 0.1 MOI and incubated at 37°C with 5% CO_2_. Cytopathic effects (CPE) were observed daily from day 1 to day 3 after inoculation. When at least 80% of the cells produce CPE, freeze–thaw 3 times and centrifuge. The supernatant was dispensed into new RNase-free tubes.

Supernatant containing unknown amounts of GETV were serially diluted 10-fold and dispensed 1 mL of diluted specimens (10^−2^, 10^−3^, 10^−4^, 10^−5^, and 10^−6^) on Vero-E6 monolayers. Incubate at 37°C with 5% CO_2_ for 3 h, washing off the supernatant, and covering the monolayer of cells with 1% carboxymethyl cellulose (CMC). After 3–5 days of incubation, when these lesions were observed to develop into plaques, the cells were fixed using 4% formaldehyde solution and crystal violet staining solution was added to enhance plaque visualization. Record the number of empty plaques per dilution and calculate the titer of GETV (PFU/mL) referring to Formula 1.


(1) $$\begin{array}{l} \mathrm{Titer of GETV}\;\left(\mathrm{in}\;\mathrm{PFU}/\mathrm{mL}\right)\\ =\frac{\mathrm{Number of plaques}}{\mathrm{Dilution factor}\times \mathrm{Volume of virus added to the well}} \end{array}$$

### Nucleic acid extraction

Mosquito specimens were stored in 2 mL tubes and centrifuged after grinding with a multi-sample tissue grinder (Shanghai Jingxin Industrial Development Co., Ltd., China). The supernatant was collected and RNA was extracted using the QIAamp Viral RNA Mini Kit (QIAGEN N.V., Germany) according to the instructions. Meanwhile, the same method was applied to extract RNA from GETV (known viral titer), CHIKV, JEV, PRRSV, AKAV, EIV, SINV, and ZIKV viral fluids and animal serum samples. The above-extracted RNA samples were stored at −80°C until use.

### Preparation of standard RNA and primer design

The whole-genomic RNA of GETV JL17/08 was serially diluted 10-fold from the stock solution to 10^−7^ in RNase-free centrifuge tubes, and stored at −80°C. The primers were designed based on the GETV *Nsp1*. All published sequences of GETV *Nsp1* were retrieved from the National Center for Biotechnology Information (NCBI) (May 2022). Sixty-one sets of GETV *Nsp1* gene sequences were aligned using MEGA X (Mega Limited, New Zealand). Then, DNASTAR (DNASTAR, Inc., United States) software was used for multiple sequence analysis to select a conserved region. A set of primers based on the sequences was designed (forward: 5′-AGCATTTTCGCATCTGGCTAC-3′) (reverse: 5′-TCTGGGTCTTCCGCACTTTT-3′), and the expected length based on this primer was 150 bp. The conserved specificity of the primers was further verified by Primer-Blast (NCBI). The primers were synthesized by Sangon Biotech (Changchun, Co., Ltd).

### Optimization of one-step RT-qPCR for GETV and establishment of standard curve

Using the extracted GETV RNA as a template, the synthesized primers were used for the detection and quantification of GETV RNA. Primer concentration (0.1–0.5 μM/l), annealing temperature (58–65°C), and number of cycles (38–42cycels) were optimized by checkerboard method. Refer to the instructions for the One-Step TB Green^®^ Prime Script™ PLUS RT-PCR Kit: 2 × One-Step TB Green RT-PCR Buffer 4 12.5 μl, TaKaRa Ex Taq HS Mix 1.5 μl, Prime Script PLUS RTase Mix 0.5 μl, PCR Forward Primer 1 μl, PCR Reverse Primer 1 μl, total RNA 2 μl, RNase-Free ddH_2_O 6.5 μl were added to prepare a total volume of 25 μl of RT-qPCR mix. The reaction conditions were Stage 1: 42°C for 5 min and 95°C for 10 s, 1 cycle, Stage 2: 95°C for 5 s and 60°C for 30 s, for each cycle, and Stage 3: 65–95°C, increment 5°C for 5 s. The RT-qPCR reaction conditions with the minimum *Cq* value, the highest fluorescence value, and the specific melting curve were selected as the most suitable reaction conditions to perform RT-qPCR amplification with 10-fold gradient diluted RNA as templates. The logarithm of viral PFUs (x-axis) was plotted against the corresponding *Cq* values (*y*-axis) to form the standard curve.

### Sensitivity, specificity, and repeatability testing

Ten-fold gradient dilutions of RNA (10^−1^–10^−7^) were used as templates for RT-qPCR and conventional RT-PCR amplification, respectively. The detection limit of qPCR was analyzed by amplification curves. Conventional PCR was first performed by reverse transcription to obtain cDNA in a total volume of 5 μl: 5 × PrimeScript RT Master Mix (Perfect Real Time) 1 μl, Total RNA 2 μl, and RNase-Free ddH_2_O 2 μl. The reaction conditions were 37°C for 15 min and 85°C for 5 s. Then, PCR Forward Primer 1 μl, PCR Reverse Primer 1 μl, 2 × Taq PCR Mix 12.5 μl, cDNA 5 μl, and ddH_2_O 5.5 μl were added to prepare a total volume of 25 μl of PCR mix, 94°C for 3 min, 94°C for 30 s, 55°C for 30s, 72°C for 1 min, 30 cycles, and 72°C for 5 min, and stored at 4°C. The detection limit of conventional PCR was obtained by agarose gel electrophoresis.

Seven viruses, CHIKV, JEV, PRRSV, AKAV, EIV, SINV, and ZIKV, were used to evaluate the specificity of RT-qPCR. Their RNA was diluted to the same concentration, with the positive control of GETV RNA and the negative control of ddH_2_O.

Using 1 × 10^4^, 1 × 10^5^, and 1 × 10^6^ PFU/mL of GETV RNA as templates to evaluate the intra- and inter-batch reproducibility of this RT-qPCR method. Each sample was replicated 3 times in the same plate and the assay was repeated 3 times in different plates at different times. On the basis of the *Cq* values of each replicate, calculated and analyzed their mean *Cq*, standard deviation (SD), and coefficient of variation (CV).

### Comparison between SYBR green I RT-qPCR and Taqman RT-qPCR

The Taqman RT-qPCR which was established according to the diagnostic techniques for animal GETV infection in the agricultural industry standards of the People’s Republic of China (draft standard for examination) was selected as the gold standard. Primers and probes were synthesized according to the sequences provided in the document. One hundred and forty-three laboratory-preserved serum samples verified as strongly positive by ELISA were tested by SYBR Green I RT-qPCR (established in this study) and Taqman RT-qPCR to analyze positive rate and the coincidence rate with consistency. The *Cq* values of the tested methods and the qualitative test results of the gold standard were recorded. The ROC curve analysis was done using Graph Pad Prism9 software (GraphPad Software Inc., CA) and SPSS 16.0 for Windows (SPSS Inc., United States). The best cutoff value that maximized the Youden index (J = Sensitivity + Specificity–1) was determined (Heuckenkamp et al., 2015). The AUC was also calculated to evaluate the accuracy of the method for diagnosing GETV (Low diagnostic value between 0.5 and 0.7, moderate between 0.7 and 0.9, and high between 0.9 and 1). *p* < 0.05 was taken as statistical significance.

### Detection of clinical samples

A total of 3,018 animal serum samples from swine, bovine, horse, sheep, and mosquito samples were collected in Heze, Jining, Yantai, Qingdao, and Binzhou in Shandong Province, China, with 1,106, 597, 667, 477, and 171 specimens per species, respectively. SYBR Green I RT-qPCR was applied to the above specimens, and the positive rate was calculated for each animal in each city.

## Results

### Establishment of the standard curve for RT-qPCR

**Figure 1:**
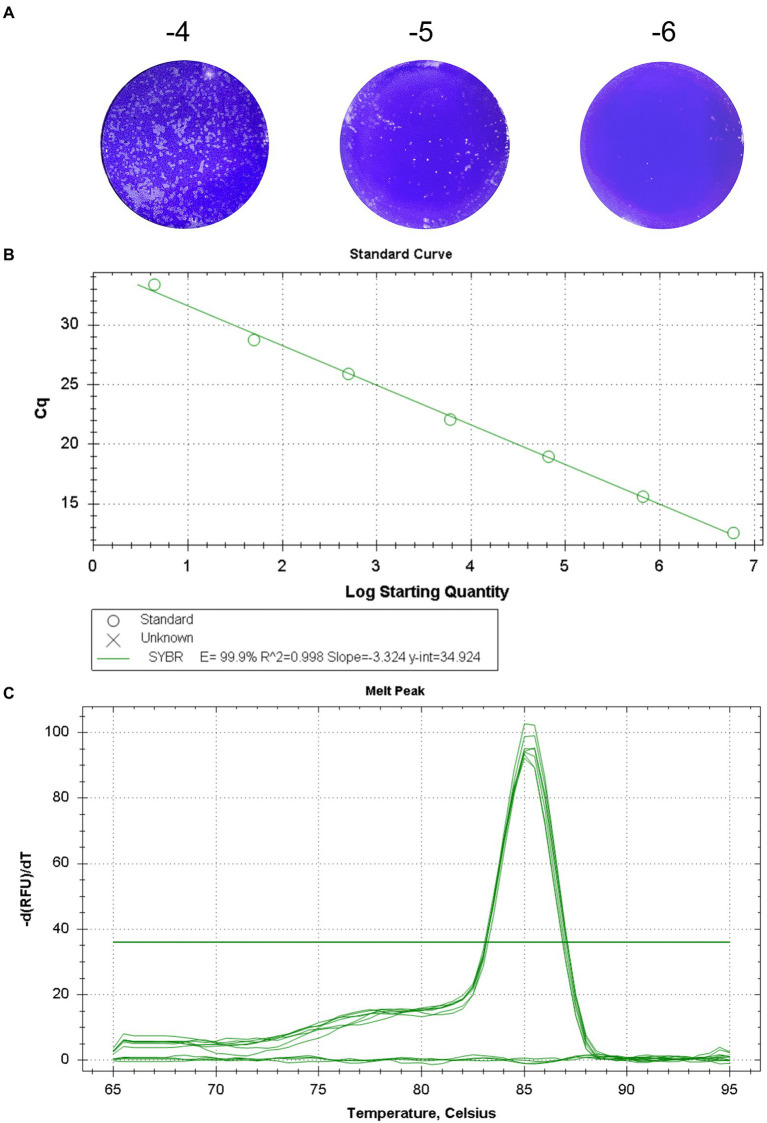
Standard curve of the real-time fluorescent quantitative PCR for GETV. **(A)** The titer of GETV was 7.73 × 10^6^ PFU/mL. **(B)** Standard curve for GETV RNA generation using tenfold serial dilutions. The concentration range of RNA dilutions is 7.73 × 10^−1^ -7.73 × 10^6^. The equation for the standard curve was *y* = −3.324 *x* + 34.924 *R*^2^ = 0.998). *y*-axis, *Cq* value; *x*-axis, log value of the viral titers corresponding to the template RNA. **(C)** The melting curve showed that all the positive samples had the same melting temperature of 85.5 ± 0.5°C and a single peak.

The titer of GETV titrated by plaque assays was 7.73 × 10^6^ PFU/mL (Figure 1A). The RT-qPCR procedure was optimized, and the optimal primer concentration was 0.4 μM, the optimal annealing temperature was 60°C, and the optimal number of cycles was 40. Standard curve was constructed using serial 10-fold gradient dilution RNA (7.73 × 10^6^ PFU/mL–7.73 × 10^−1^ PFU/mL). The linear regression equation for the standard curve was *y* = −3.324*x* + 34.924. The correlation coefficient *R*^2^) was 0.998 and the amplification efficiency (E) was 99.9% (Figure 1B). Analysis of the melting curve showed that all the positive samples had the same melting temperature of 85.5 ± 0.5°C forming a single peak, with no primer dimer curve (Figure 1C).

### Sensitivity, specificity, and reproducibility of RT-qPCR detection

**Figure 2:**
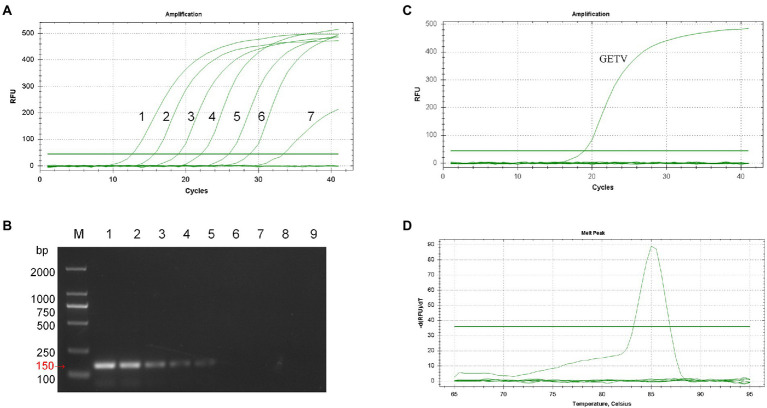
Sensitivity and specificity analysis of SYBR Green I RT-qPCR for GETV. **(A)** Amplification curve. 1–7: 7.73 × 10^6^ PFU/mL–7.73 × 10^−0^ PFU/mL. **(B)** Conventional PCR sensitivity detection. M: DL2000 marker; 1–8: 7.73 × 10^6^ PFU/mL–7.73 × 10^–1^ PFU/mL; 9: non-template-control. **(C)** Only GETV had an amplification curve. JEV, PRRSV, AKAV, EIV, SINV, ZIKV, and negative controls had no fluorescence signal amplification. **(D)** Only GETV had a specific melting peak at 85.5 ± 0.5°C, with no non-specific products.

Co-analyzing the standard curve and the amplification curve, we concluded that the detection limit of SYBR Green I RT-qPCR was 7.73 × 10^0^ PFU/mL (Figure 2A), and the detection limit of RT-PCR was 7.73 × 10^2^ PFU/mL (Figure 2B). The detection limit of RT-qPCR showed 100-fold higher than that of RT-PCR. The specificity of the established RT-qPCR assay was validated by GETV, CHIKV, JEV, PRRSV, AKAV, EIV, SINV, and ZIKV. The results showed that only GETV was specifically amplified (Figure 2C), with only one melting point peak on the curve, and melting curve analysis did not reveal non-specific products such as amplified primer dimers (Figure 2D). Agarose gel electrophoresis was performed on all samples, and no specific PCR products were found. The amplified product consisted of a 150 bp GETV *Nsp1* fragment verified by gene sequencing and BLAST comparison. The results of intra- and inter-batch reproducibility of this RT-qPCR method are shown in Table 1. The intra-assay CV ranged from 0.45% to 0.57% and the inter-assay CV ranged from 0.86% to 1.05%.

**Table 1:** Intra- and inter-assay reproducibility analysis of SYBR Green I RT-qPCR for GETV.

Viral titers (PFU/mL)	Intra-assay			Inter-assay		
	Mean(Ct)	*SD*	CV(%)	Mean(Ct)	*SD*	CV(%)
7.73 × 10^4^	19.73	0.11	0.57	19.91	0.17	0.86
7.73 × 10^5^	16.61	0.08	0.45	16.76	0.18	1.05
7.73 × 10^6^	13.57	0.07	0.52	13.69	0.13	0.91

### Coincidence between SYBR green I RT-qPCR and Taqman RT-qPCR for GETV

**Figure 3:**
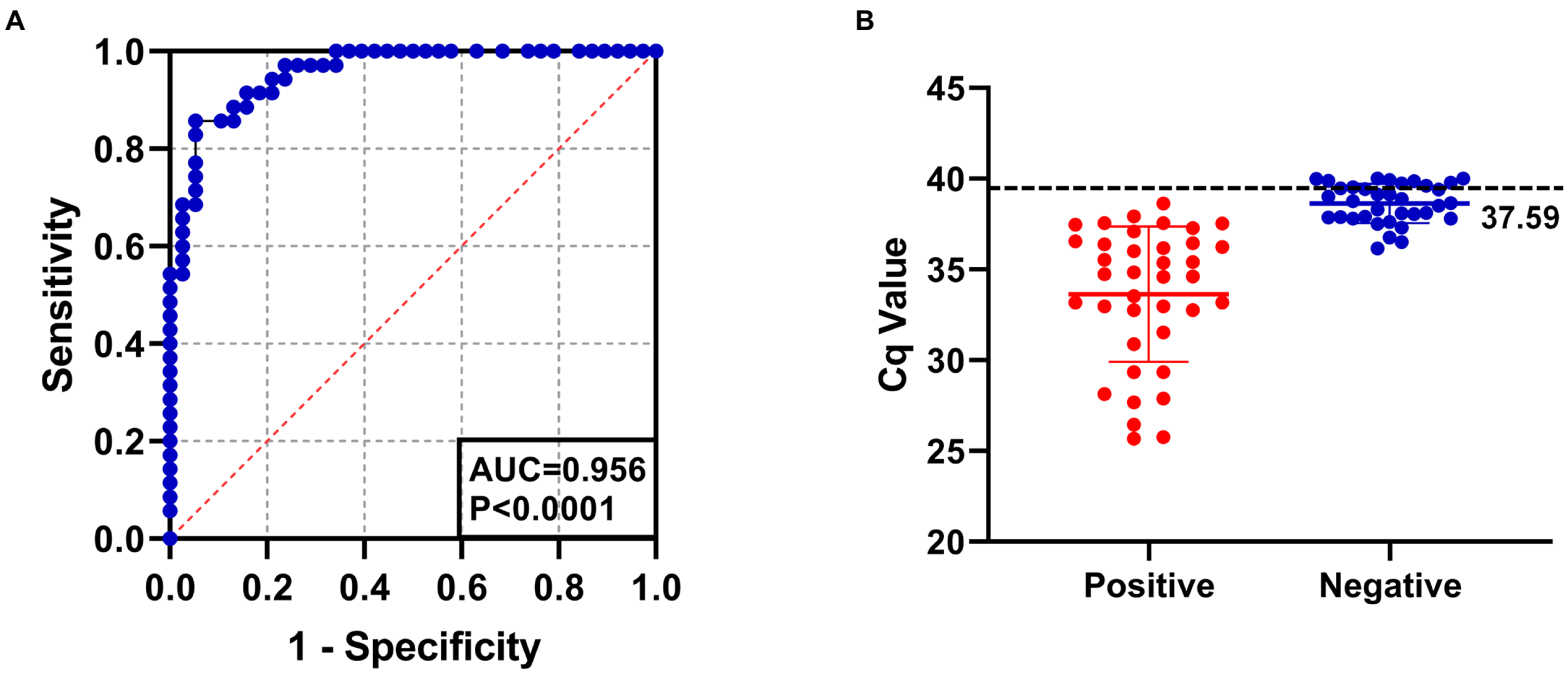
**(A)** The ROC curve analysis for assessing the diagnostic accuracy of SYBR Green I RT-qPCR assay. AUC: 0.956, *p* < 0.0001. **(B)** The best cutoff value of SYBR Green I RT-qPCR assay was 37.59.

The positive rate of 143 clinical serum samples kept in the laboratory that tested strongly positive by ELISA was 29.4% by SYBR Green I RT-qPCR method and 26.6% by Taqman RT-qPCR method. In addition, the established SYBR Green I RT-qPCR with 94.7% sensitivity and 94.3% specificity had a high total coincidence rate (94.4%) and a decent consistency (Kappa value 0.863) with Taqman RT-qPCR (Table 2). The ROC curve of SYBR Green I RT-qPCR is shown in Figure 3A and the corresponding AUC was 0.956 (*p* < 0.0001), which proved that this method has high diagnostic value. The best *Cq* cutoff value was calculated to be 37.59 (Figure 3B), with a sensitivity of 85.7% and specificity of 94.7%.

**Table 2:** Comparison of SYBR Green I RT-qPCR and Taqman RT-qPCR.

		Taqman RT-qPCR		
		Positive	Negative	Total
SYBR Green I RT-qPCR	Positive	36	6	42
	Negative	2	99	101
	Total	38	105	143
Coincidence rate		94.4% (135/143)		
Kappa		0.863		

### Detection of clinical specimens by SYBR green I RT-qPCR

**Figure 4:**
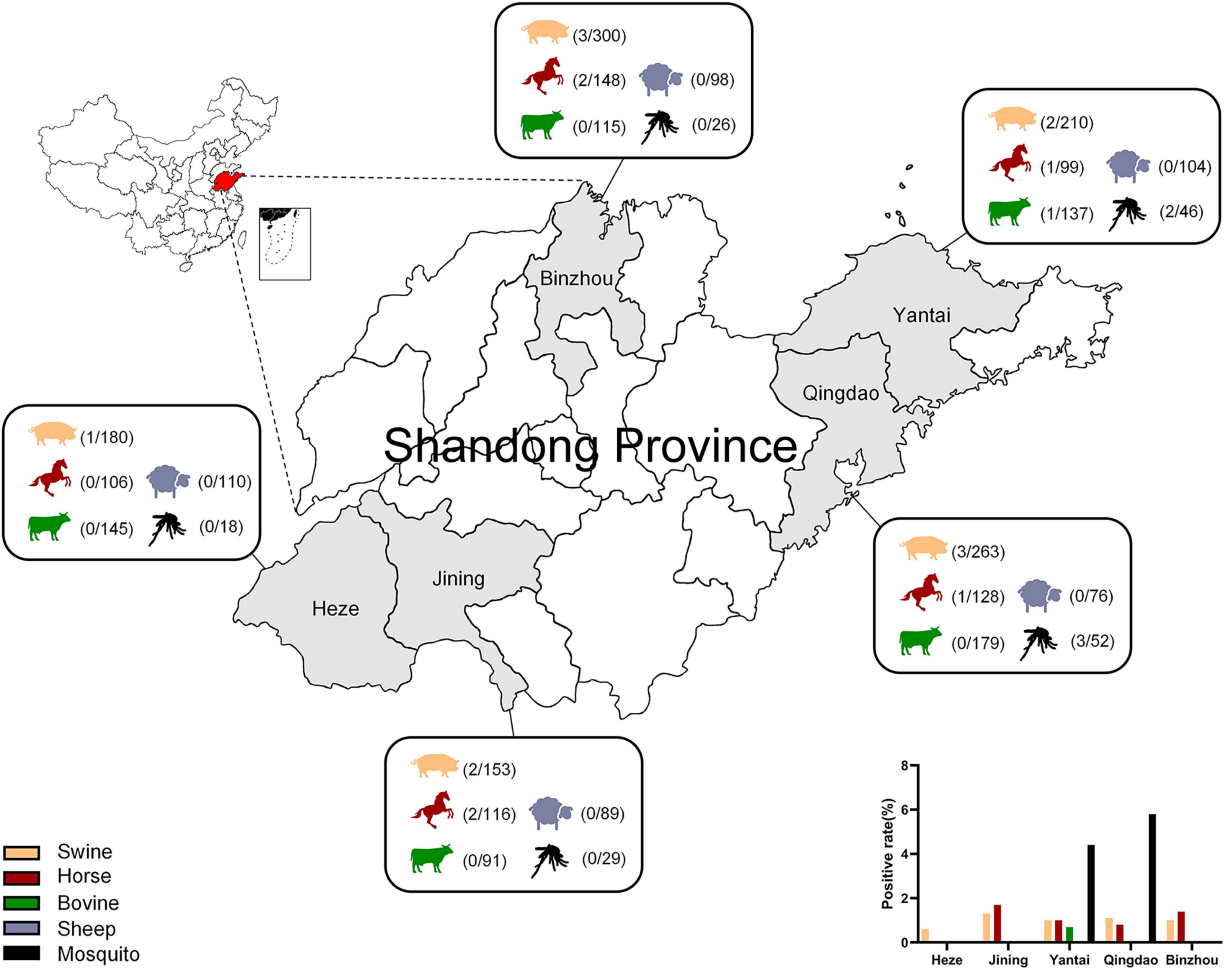
Serum samples of swine, bovine, horse, sheep, and mosquito samples from Heze, Jining, Yantai, Qingdao, and Binzhou in Shandong Province, China, were collected for RT-qPCR and the positivity rate of each animal was calculated.

The results showed that the positive rates of GETV nucleic acid in Shandong (Heze, Jining, Yantai, Qingdao, Binzhou) were 1%, 1%, 0.2%, 0%, and 3% in swine, horses, bovine, sheep, and mosquitoes, respectively (Table 3; Figure 4). The positive rates of swine in these five cities ranged from 0.6% to 1.3% and that of horses in Jining was the biggest with 1.7%, while no GETV was detected in Heze. Interestingly, GETV from bovine samples was only detected in Yantai with 0.7% positive rate, whereas positive samples from sheep were not detected in any of the five cities. Mosquitoes, as an important vector of GETV, were found to carry GETV merely in Yantai (4.4%) and Qingdao (5.8%). To further prove the above results, two strong positive RT-qPCR products were taken from each of the above five animal samples for sequencing and BLAST analysis, and the results showed that all of them were GETV *Nsp1* gene sequences. The SYBR Green I RT-qPCR and sequencing results were in 100% agreement.

**Table 3:** Positive rate of GETV in five species of animals in five cities in Shandong Province.

Species	Total (%)	City (%)				
		Heze	Jining	Yantai	Qingdao	Binzhou
Swine	11/1106 (1)	1/180 (0.6)	2/153 (1.3)	2/210 (1)	3/263 (1.1)	3/300 (1)
Horse	6/597 (1)	0/106 (0)	2/116 (1.7)	1/99 (1)	1/128 (0.8)	2/148 (1.4)
Bovine	1/667 (0.2)	0/145 (0)	0/91 (0)	1/137 (0.7)	0/179 (0)	0/115 (0)
Sheep	0/477 (0)	0/110 (0)	0/89 (0)	0/104 (0)	0/76 (0)	0/98 (0)
Mosquito	5/171 (3)	0/18 (0)	0/29 (0)	2/46 (4.4)	3/52 (5.8)	0/26 (0)

## Discussion

As a mosquito-borne disease, GETV mainly causes abortion in pregnant sows and generalized rash and leg edema in horses. Natural infection of GETV has been found to occur in a variety of vertebrates, including humans. Moreover, as an effective tool for disease prevention and control, the commercialized vaccine of GETV was only successfully developed and applied in livestock industry in Japan (Tajima et al., 2014). With its wide geographic distribution and strong host adaptation, as well as the lack of effective vaccines in other countries, GETV has become an increasingly serious threat to public safety and the livestock economy. Therefore, there is an urgent requirement to establish a rapid assay to characterize and quantify the infectious GETV with sensitivity and specificity.

At present, RT-qPCR methods are currently available for detecting GETV, including TaqMan and SYBR Green I. The probe approach in TaqMan has shown higher sensitivity than the SYBR-Green method. However, studies have shown that virus escape has recently been observed in various strains of the Taqman-type method (Tam et al., 2009; Leifer et al., 2010). In addition, hybridization errors between probe and template can be attributed to nucleotide changes in the sequence of the viral preparation (King et al., 2006). In contrast, the SYBR Green I method not only avoids the risk of probe mismatches (Acevedo et al., 2013; Zhu et al., 2013), but its melting curve can also help to identify mutations occurring in the PCR target region to compensate for other molecular methods.

In this study, SYBR Green I RT-qPCR assay for GETV was successfully established. The linearity of the standard curve was excellent, with a correlation coefficient (*R*^2^) of 0.998. The amplification efficiency (*E*) was 99.9%, complied with the technical requirement of within 105%. The minimum detection limit was 7.73 PFU/mL, which was 100 times more sensitive than that of the common RT-PCR method, and meanwhile, slightly more sensitive than that of the similar method by Dong, with 1 × 10^1^ PFU/mL (Dong et al., 2012).

Most of the published studies on GETV RT-qPCR assays have established standard curves with recombinant plasmids that can be converted to copy number by the formula to quantify the virus in copies/mL, which is the number of genes or plasmids contained in a unit volume. Differently, in this study, the standard curve was established directly with RNA of GETV that was titrated by viral plaque assay whose unit is PFU/mL, which refers to the number of invasive viral particles contained in each milliliter of sample test solution. This measurement is also gradually being used in the literature related to *Alphavirus* (McMillen et al., 2018; Fumagalli et al., 2021).

The turnover in measurement standards may be explained by the fact that the viral plaque assay is the gold standard for detecting viral content and infectivity. Its visualization and determination of viral infectivity cannot be replaced by many other assays at the molecular level. Some studies have verified that the viral titer of the virus has a strong linear correlation with the RNA copy number of the viral genome (*R*^2^ = 0.957), and the RNA copy number is larger than the viral titer determined by the plaque assay. The ratios of RNA copy number to viral titer range from 2 to 3 log units or more (Houng et al., 2000; Garcia et al., 2001; Bae et al., 2003; Guillaume et al., 2004). This difference can be attributed to the accumulation of defective viral particles and the release of unpackaged viral RNA from ruptured infected cells during viral infection and self-replication (Guillaume et al., 2004). Furthermore, previous studies using copy number as the unit of measurement had found that positive result of qPCR did not necessarily mean the presence of virus transmission (La Scola et al., 2020; Shrestha et al., 2020). In conclusion, in quantifying the number of virus particles that are still infectious, the plaque-forming unit PFUs of the virus has more clinically significant than the copy number of the virus. Compared to plasmids, the use of RNA as a standard sample is also less likely to produce aerosol contamination, which is a problematic issue in nucleic acid detection experiments.

At present, the established RT-qPCR methods for the detection of GETV are directed for mosquito or swine serum. In contrast, GETV is an arbovirus with a broad spectrum of infection, and an RT-qPCR assay that can be applied to multiple species of swine, horses, bovines, sheep, and mosquitoes is of more practical application. One study performed multiple sequence alignment analysis and phylogenetic analysis of 45 whole-genome sequences, 71 *E2*, 47 *E1*, 61 *Nsp1,* and 57 *Cap* gene sequences of GETV from NCBI and found that the *Nsp1* gene was the most stable and had the lowest mutation rate during the evolution of GETV transmission (Shi et al., 2021). In this study, specific primers were designed based on the conserved sequence of *Nsp1* of GETV, and after optimization, the method was found to have excellent specificity, with a specific melting peak at 85.5 ± 0.5°C. The specificity test only detects significant fluorescence signal for GETV, without cross-reactivity with CHIKV, JEV, PRRSV, AKAV, EIV, SINV, and ZIKV. The intra- and inter-group reproducibility results showed that the method has good reproducibility and stability. The ROC curve of SYBR Green I RT-qPCR showed the high diagnostic value of this method. For the first time, the best cutoff value was determined for qualitative detection of GETV clinical samples, providing a more reliable method for RT-qPCR to define negative or positive clinical samples.

Results of the epidemiological investigation of GETV nucleic acid in Shandong Province, China, showed that the total positive detection rates in swine, horses, bovines, sheep, and mosquitoes were 1%, 1%, 0.2%, 0%, and 3%, respectively. The positive sera for GETV in swine were detected in five cities of Shandong Province, namely Heze, Jining, Yantai, Qingdao, and Binzhou. Positive sera from horses were detected in four cities except Heze. It is speculated that this may be related to the fact that swine and horses are the main amplification hosts of GETV. Nucleic acid-positive sera from cattle were detected only in a free-range cattle farm in Yantai. Sheep had no positive cases in any of the five cities, and no positive cases of GETV in sheep have been reported so far. What calls for special attention is that the positive specimens of mosquitoes were only detected in two coastal cities, Yantai and Qingdao, and showed a high positive rate, suggesting that coastal cities are denser with mosquitoes and need to do a more effective elimination of mosquitoes in farms. The positive detection rates of GETV are basically consistent with the results of the reported GETV prevalence survey (Dong et al., 2012; Lu et al., 2019; Ren et al., 2020; Zhang et al., 2022) but only differed significantly from the positive detection rate of RT-qPCR established by Xia which reached 34.5% in swine serum (Xia et al., 2021). We analyze that this may be related to the mosquito eradication and prevention measures of different farms. Some positive products of RT-qPCR were selected for sequencing and BLAST analysis, and the compliance rate between RT-qPCR and sequencing results was 100%.

## Conclusion

In this study, we innovatively developed a specific, sensitive, repeatable, and economical RT-qPCR method combined with plaque assay for the detection of infectious GETV. And successfully applied to the epidemiological investigation of swine, horses, bovines, sheep, and mosquitoes, revealing the epidemic status of GETV in Shandong Province. The establishment of this method also laid the foundation for the subsequent research of GETV.

## Data availability statement

The raw data supporting the conclusions of this article will be made available by the authors, without undue reservation.

## Ethics statement

Ethical review and approval was not required for the animal study because No animal testing was performed. Written informed consent was obtained from the owners for the participation of their animals in this study.

## Author contributions

NJ, NS, and HL conceptualized the study. XC performed the study and drafted the manuscript. XQ coordinated the analysis of the data and drafted the manuscript. ZH, HZ, PW, XZ, YX, GZ, and WZ participated in the design of the study and contributed the sample. All authors contributed to the article and approved the submitted version.

## Funding

This work was supported by the National Key Research and Development Program of China (2021YFC2301704) and CAMS Innovation Fund for Medical Sciences (2020-12M-5-001).

## Conflict of interest

The authors declare that the research was conducted in the absence of any commercial or financial relationships that could be construed as a potential conflict of interest.

## Publisher’s note

All claims expressed in this article are solely those of the authors and do not necessarily represent those of their affiliated organizations, or those of the publisher, the editors and the reviewers. Any product that may be evaluated in this article, or claim that may be made by its manufacturer, is not guaranteed or endorsed by the publisher.
